# London Prices of Drugs.—Dec. 24

**Published:** 1814-01

**Authors:** 


### LONDON PRICES OF DRUGS.—Dec. 24.

*(To be continued regularly.)*

| | £. | s. | d. | £. | s. | d. | per |
|---|---|---|---|---|---|---|---|
| Aloes Barbadoes, from | 21 | 0 | 0 to | 22 | 0 | 0 | C. |
| —— Cape | 9 | 10 | 0.. | 10 | 0 | 0 | — |
| —— Succotrina | 20 | 0 | 0.. | 0 | 0 | 0 | — |
| —— Epatica or E.I. | 10 | 0 | 0.. | 12 | 0 | 0 | — |
| Angelica Root | 15 | 0 | 0.. | 16 | 10 | 0 | — |
| Alkanet Root | 9 | 15 | 0.. | 10 | 10 | 0 | — |
| Antimony Crude | 3 | 15 | 0.. | 4 | 6 | 0 | — |
| Aqua Fortis | S. | 0 | 8.. | D. | 1 | 1 | lb |
| Arrow Root fine | 0 | 2 | 0.. | 0 | 3 | 6 | — |
| —— ordinary | 0 | 1 | 0.. | 0 | 1 | 6 | — |
| Arsenic Red | 6 | 10 | 0.. | 6 | 15 | 0 | C. |
| —— White | 3 | 10 | 0.. | 3 | 12 | 0 | — |
| Balsam Capivi | 0 | 5 | 6.. | 0 | 0 | 0 | lb. |
| —— Peru | 0 | 19 | 0.. | 1 | 0 | 0 | — |
| —— Tolu | 0 | 12 | 0.. | 0 | 0 | 0 | — |
| Bark Jesuits Com. | 0 | 3 | 6.. | 0 | 4 | 0 | — |
| —— Second | 0 | 6 | 0.. | 0 | 7 | 0 | — |
| —— Quil or best | 0 | 8 | 0.. | 0 | 10 | 0 | — |
| —— Red | 0 | 7 | 0.. | 0 | 10 | 0 | — |
| —— Yellow | 0 | 4 | 6.. | 0 | 5 | 0 | — |
| Borax refined E.I. | 5 | 10 | 0\.. | 6 | 0 | 0 | C. |
| —— English | 0 | 2 | 8.. | 0 | 0 | 0 | lb. |
| —— unrefined or Tinc. | 6 | 0 | 0.. | 0 | 0 | 0 | C. |
| Camphire refined | 0 | 6 | 9.. | 0 | 0 | 0 | lb. |
| —— unrefined | 24 | 0 | 0.. | 25 | 0 | 0 | C. |
| Cantharides | 0 | 11 | 6.. | 0 | 11 | 9 | — |
| Cardamoms (best) | 0 | 7 | 6.. | 0 | 0 | 0 | lb. |
| Cassia Buds | 12 | 0 | 0.. | } | 0 | 0 | C. |
| —— Fistula W.I. | 12 | 0 | 0.. | } in bond | | | — |
| —— Lignia | 12 | 0 | 0.. | } | 0 | 0 | — |
| Castorum American | 2 | 14 | 0.. | 2 | 16 | 0 | lb. |
| —— Russia | 11 | 0 | 0.. | 0 | 0 | 0 | — |
| Castor Oil per bottle 1½ lb. | 0 | 3 | 9.. | 0 | 4 | 3 | bo |
| Coculus Indicus | 3 | 0 | 0.. | 0 | 0 | 0 | C. |
| Colocynth Turkey | 0 | 5 | 0.. | 0 | 5 | 6 | lb. |
| Columbo Root | 2 | 15 | 0.. | 3 | 0 | 0 | C. |
| Cream of Tartar | 8 | 10 | 0.. | 9 | 5 | 0 | — |
| Gallangal East-India | 5 | 0 | 0.. | 0 | 0 | 0 | C. |
| Gentian Root | 4 | 10 | 0.. | 0 | 0 | 0 | — |
| Ginsang | 0 | 1 | 6.. | 0 | 0 | 0 | lb. |
| Grains of Guinea | 5 | 10 | 0.. | 6 | 0 | 0 | C. |
| Gum Ammo. Drop | 30 | 0 | 0.. | 0 | 0 | 0 | — |
| —— Lump | 8 | 0 | 0.. | 9 | 0 | 0 | — |
| —— Animi | 3 | 10 | 0.. | 7 | 10 | 0 | — |
| —— Arabic E.I. | 2 | 0 | 0.. | 4 | 0 | 0 | — |
| —— Turkey Fine | 8 | 10 | 0.. | 9 | 9 | 0 | — |
| —— Barbary | 6 | 0 | 0.. | 7 | 0 | 0 | — |
| —— Assafœtida | 10 | 0 | 0.. | 15 | 0 | 0 | — |
| —— Benjamin | 8 | 10 | 0.. | 35 | 0 | 0 | — |
| —— Cambogium | 20 | 0 | 0.. | 26 | 0 | 0 | — |
| —— Copal scraped | 0 | 2 | 0.. | 0 | 3 | 6 | lb. |
| —— Galbanum | 16 | 0 | 0.. | 20 | 0 | 0 | C. |

| | £. | s. | d. | £. | s. | d. | per |
|---|---|---|---|---|---|---|---|
| Gum Guaiacum, from | 0 | 2 | 6 to | 0 | 3 | 0 | lb. |
| —— Mastic | 0 | 4 | 6.. | 0 | 4 | 9 | — |
| —— Myrrh | 15 | 0 | 0.. | 18 | 0 | 0 | C. |
| —— Olibanum | 6 | 0 | 0.. | 7 | 0 | 0 | — |
| —— Oppoponax | 80 | 0 | 0.. | 0 | 0 | 0 | — |
| —— Sandrac | 6 | 10 | 0.. | 8 | 0 | 0 | — |
| —— Seneca Garbled | 5 | 2 | 0.. | 5 | 5 | 0 | — |
| —— Tragacanth | 36 | 0 | 0.. | 38 | 0 | 0 | — |
| Jalap | 0 | 5 | 0.. | 0 | 5 | 3 | lb. |
| Ipecacuanha | 0 | 16 | 0.. | 0 | 0 | 0 | — |
| Isinglass Book | 0 | 12 | 0.. | 0 | 13 | 0 | — |
| —— Leaf | 0 | 12 | 0.. | 0 | 13 | 0 | — |
| —— Long Staple | 0 | 12 | 0.. | 0 | 13 | 0 | — |
| —— Short Staple | 0 | 12 | 0.. | 0 | 13 | 0 | — |
| Manna Flakey | 0 | 8 | 0.. | 0 | 8 | 6 | — |
| —— Sicily in Sorts | 0 | 5 | 0.. | 0 | 0 | 0 | — |
| Musk China | 0 | 18 | 0.. | 1 | 0 | 0 | oz. |
| Nux Vomica | 3 | 0 | 0.. | 3 | 10 | 0 | C. |
| Oil of Vitriol | 0 | 0 | 3¼ | 0 | 0 | 0 | lb. |
| Opium East-India | 0 | 19 | 6.. | 1 | 0 | 0 | — |
| —— Turkey | 1 | 2 | 0.. | 0 | 0 | 0 | — |
| Pink Root | 0 | 5 | 0.. | 0 | 0 | 0 | — |
| Quicksilver | 0 | 4 | 6.. | 0 | 4 | 8 | — |
| Rhubarb East-India | 0 | 3 | 0.. | 0 | 5 | 0 | — |
| —— Russia | 0 | 14 | 0.. | 0 | 0 | 0 | — |
| Saffron Spanish | 1 | 10 | 0.. | 1 | 15 | 0 | — |
| —— French | 1 | 10 | 0.. | 0 | 0 | 0 | — |
| Sago | 6 | 0 | 0.. | 6 | 10 | 0 | C. |
| Sal. Ammoniac | 10 | 10 | 0.. | 11 | 0 | 0 | — |
| Sarsaparilla | 0 | 3 | 0.. | 0 | 3 | 6 | lb. |
| Sassafras | 8 | 0 | 0.. | 9 | 0 | 0 | T. |
| Scammony Aleppo | 1 | 3 | 0.. | 1 | 5 | 0 | lb. |
| —— Smyrna | 0 | 10 | 0.. | 0 | 12 | 0 | — |
| Senna | 0 | 3 | 0.. | 0 | 0 | 0 | — |
| Seeds Anni Alicant | 6 | 0 | 0.. | 6 | 10 | 0 | C. |
| —— Coriander English | 1 | 10 | 0.. | 1 | 16 | 0 | — |
| —— Cummin | 6 | 0 | 0.. | 7 | 0 | 0 | — |
| —— Fenugreek | 1 | 15 | 0.. | 2 | 0 | 0 | — |
| Shellack | 7 | 0 | 0.. | 8 | 8 | 0 | — |
| Sticklack | 6 | 0 | 0.. | 8 | 0 | 0 | — |
| Snake Root | 0 | 16 | 0.. | 0 | 0 | 0 | lb. |
| Soap Castile or Spanish | 10 | 0 | 0.. | 11 | 0 | 0 | C. |
| Spermaceti refined | 0 | 2 | 7.. | 0 | 0 | 0 | lb. |
| Tamarinds West-India | 8 | 10 | 0.. | 9 | 0 | 0 | C. |
| Tapioca Lisbon | 0 | 0 | 8.. | 0 | 1 | 0 | lb. |
| Turmeric Bengal | 3 | 3 | 0.. | 0 | 0 | 0 | C. |
| —— China | 4 | 5 | 0.. | 4 | 15 | 0 | — |
| —— West-India | 6 | 10 | 0.. | 7 | 0 | 0 | — |
| Verdigris Wet | 0 | 3 | 6.. | 0 | 0 | 0 | lb. |
| —— Dry | 0 | 5 | 10.. | 0 | 0 | 0 | — |
| —— Crystallized | 0 | 8 | 6.. | 0 | 8 | 9 | — |
| Vitriol Roman | 0 | 0 | 7.. | 0 | 0 | 0 | — |
| —— Foreign white | 3 | 10 | 0.. | 3 | 15 | 0 | C. |

*Price of Vials per Gross.*—8 oz. 70s.—6 oz. 58s.—4 oz. 47s.—3 oz. 43s.—2 oz. 36s.—1 oz. 30s.—½ oz. 24s.

### OBSERVATIONS.

At recent Sales of Merchandize by Auction, *Messrs.* Piston *and* Co. sold 26 Chests of Yellow Bark, 3s. 1d. to 4s. per lb.—3 Chests Gum Benjamin, duty paid, 29l. 16s. to 30l. 15s. per Cwt.—5 Chests ditto, in bond, 16l. 10s. per Cwt.—2 Chests Gum Myrrh, 20l. 15s. per Cwt.—11 Barrels White Arsenic, 68s to 69s. per Cwt.—18 Bags of Turmeric, 92s. to 100s. per Cwt.—2 Casks Gum Arabic, 7l. 15s. per Cwt.

